# Myosin 1f-mediated activation of microglia contributes to the photoreceptor degeneration in a mouse model of retinal detachment

**DOI:** 10.1038/s41419-021-03983-3

**Published:** 2021-10-09

**Authors:** Yimin Wang, Xiaohuan Zhao, Min Gao, Xiaoling Wan, Yinong Guo, Yingying Qu, Yuhong Chen, Tong Li, Haiyun Liu, Mei Jiang, Feng Wang, Xiaodong Sun

**Affiliations:** 1 Shanghai General Hospital, Shanghai Jiao Tong University School of Medicine, Shanghai, China; 2 National Clinical Research Center for Eye Disease, Shanghai, China; 3 Shanghai Key Laboratory of Ocular Fundus Diseases, Shanghai, China; 4 Shanghai Engineering Center for Visual Science and Photomedicine, Shanghai, China; 5 Shanghai Institute of Immunology, Translational Medicine Center, Shanghai General Hospital, State Key Laboratory of Oncogenes and Related Genes, Shanghai Jiao Tong University School of Medicine, Shanghai, China

**Keywords:** Neuroimmunology, Retina

## Abstract

Photoreceptor death and neurodegeneration is the leading cause of irreversible vision loss. The inflammatory response of microglia plays an important role in the process of neurodegeneration. In this study, we chose retinal detachment as the model of photoreceptor degeneration. We found Myosin 1f was upregulated after retinal detachment, and it was specifically expressed in microglia. Deficiency of myosin 1f protected against photoreceptor apoptosis by inhibiting microglia activation. The elimination of microglia can abolish the protective effect of myosin 1f deficiency. After stimulation by LPS, microglia with myosin 1f deficiency showed downregulation of the MAPK and AKT pathways. Our results demonstrated that myosin 1f plays a crucial role in microglia-induced neuroinflammation after retinal injury and photoreceptor degeneration by regulating two classic inflammatory pathways and thereby decreasing the expression of inflammatory cytokines. Knockout of myosin 1f reduces the intensity of the immune response and prevents cell death of photoreceptor, suggesting that myosin 1f can be inhibited to prevent a decline in visual acuity after retinal detachment.

## Background

The loss of photoreceptors and retinal function disorder is the feature of photoreceptor degeneration, which leads to irreversible vision loss [1]. Pathogenesis is involved in many retinal diseases, including retinal detachment, retinitis pigmentosa, and age-related macular degeneration. In particular, retinal detachment is a kind of disease that photoreceptors lose nutritional support after being separated from the retinal pigment epithelium (RPE) layer and choroidal vessels, which leads to the death of photoreceptors [2]. Rhegmatogenous retinal detachment (RRD) is the most common form of retinal detachment (RD), with an incidence of 13 per 100,000 persons annually [3]. Although surgery can reattach the retina with a high success rate, a small portion of patients still experience vision loss due to photoreceptor death [4, 5]. Therefore, discovering the mechanisms of the process of cell death is crucial to neuroprotection and intervention.

Although many death effectors have been discovered and targeted to prevent the loss of photoreceptors, little progress on rescuing photoreceptor function after retinal injury [6]. Recently, transcriptome analysis has revealed that inflammatory responses play an important role in the process of photoreceptor degeneration [1, 7]. Many chemokines—such as TNF-α, IL-1β, IL-6, IL-8, and MCP-1—can reach a high level after 1 h following retinal detachment [8, 9]. These cytokines can activate microglia and recruit macrophages in the subretinal space [1]. In the meantime, the activated microglia can release MCP-1, which contributes to the increased expression of MCP-1 in Müller cells and macrophages [10]. The positive feedback aggravates the immune response and maintains a high level of neuroinflammation [1, 11], which can be harmful to photoreceptors [2].

Microglia plays a key role in the inflammatory feedback loop, which is a potential therapeutic target for neuroprotection in retinal degeneration. Activated microglia can express TNF-α and IL-1β, which are widely implicated in retinal degenerative disease. TNF-α can combine with TNF receptors 1 and 2, which are distributed on the membranes of neurons, leading to cytotoxicity by causing mitochondrial dysfunction and oxidative stress. IL-1β is able to combine with interleukin receptors and leads to cell death in a manner similar to TNF-α signaling [12, 13]. Meanwhile, it secretes MCP-1 to recruit and activates astrocytes and immune cells from circulation, aggravating the immune response [1, 11, 14]. Thus, it is important to discover the mechanisms by which microglia affect this loop in order to potentially inhibit the overactivation of microglia and the release of toxic cytokines. Mitogen-activated protein kinases (MAPKs) regulates the expression of pro-inflammatory cytokines such as TNF-α, IL-1β, and IL-6 [15]. Activated protein kinase B (PKB), also named AKT, can regulate inflammatory response by downstream factors [16]. However, the upstream mechanism is not entirely understood.

The myosin family, as a component of the cytoskeleton, has been reported to play a crucial role in cell signaling [17, 18]. Class 1 myosins have been revealed to be key components during pinocytosis [19], phagocytosis [20], cell motility [21], and secretion [22]. Therefore, the exploration of myosin function in microglia can lead to a better understanding of pathogenesis after retinal detachment. In this study, we have proven that myosin 1f is involved in the activation of microglia by regulating the MAPK and AKT pathways in mouse models of photoreceptor degeneration.

## Methods

### RNA-seq, sequencing data extraction, and bioinformatics analysis

The sequencing data (GSE28133) were downloaded from GEO, which is a public database of chips and microarrays. The data contain 38 human retinal samples, including 19 samples from RD patients and 19 control samples without RD.

Four pooled detached retinas were collected as one sample from both WT and myosin −/− mice at day 3. The sequencing platform is from illumine HiSeq 2500 system.

The data were normalized first, then DEGs were analyzed by a limma algorithm using the R programming language. The log fold change cutoff and adjusted *P* value or *P* value were set as 1.5 and 0.05, respectively. Points without gene symbols were removed.

A GO enrichment analysis of DEGs was obtained using the online tool DAVID (https://david.ncifcrf.gov/home.jsp, version 6.8). The bubble maps were drawn using Hmisc and ggplot2 via the R programming language. The GSEA analysis was conducted using GSEA_4.0.1 software.

### Retinal-detachment model animals

Myosin 1f−/− mice were purchased from Jackson Laboratory, then bred in the Shanghai General Hospital animal facility. All animal experiment protocols were in agreement with the Statement of the Association for Research in Vision and Ophthalmology for biomedical research. The animals were randomly assigned into two groups. The sample size is estimated based on the sum of the minimum sample sizes required for each experiment. All the measurements of animal models were taken blindly.

We used 1% atropine sulfate oculentum (Santen, Japan) and 0.5% tropicamide (Santen, Japan) on the ocular surface of mice to dilate the pupil. We applied 0.4% oxybuprocaine eye drops (Santen, Japan) as surface anesthesia. The mice were anesthetized using isoflurane gas (1.5% mixed with 50% air and 50% O_2_). We applied 0.3% of ofloxacin oculentum (Shanghai, China) as a magnifying lens to obtain a clearer view.

The RD model was conducted as previously described [23, 24]. The sclera was exposed and punctured at 2 mm posterior to the limbus with a 34-G needle. The vitreous humor was slowly aspirated with a 34-G glass needle until the retina separated spontaneously from the underlying RPE layer. Then the 34-G needle tip was inserted into the subretinal space through the same scleral hole, and sodium hyaluronate (HA, Shanghai, China) was gently injected. The fundus was observed and injection was halted after the retina was detached in every quadrant. The scleral hole was then sealed using cyanoacrylate surgical glue to prevent HA leakage. Finally, tobramycin and dexamethasone ointments (Alcon, USA) were applied to the ocular surface to prevent infection. The eyes with ocular infection would be excluded from further experiments.

### Immunofluorescence

The eyeballs were fixed in 4% paraformaldehyde and cut to a thickness of 10 μm to fabricate eye sections. We removed the chamber and kept the entire retina to prepare for the retinal stretched preparation after fixation.

We stained for the following antibodies: IBA1 (1:1000, Wako, 019–19741), F4/80 (1:1000, Abcam, 6640), GFAP (1:1000, Abcam, 4674), Opsin (Sigma), Tuj1 (Abcam), and myosin 1f (1:1000, Abcam, ab197215). The immunofluorescence was observed under a confocal microscope (Leica TCS SP8 confocal 137 microscope, Germany) and quantitatively analyzed using ImageJ software (Fiji, NIH, USA).

### HE stains and ONL thickness

After being fixed in 4% paraformaldehyde, the eyeballs were embedded in paraffin and sectioned into 10-μm slices. The eye sections were stained with hematoxylin and eosin. We measured ten points of thickness within the outer nuclear layer (ONL) on one section with the same spacing distance [23, 24], then calculate the average thickness of each eyeball. We compared the average thickness of different groups. The “n number” in the figure is the number of eyeballs. The measurements were taken using ImageJ software (Fiji, NIH, USA).

### TUNEL assay

In-Situ Cell Death Detection Kits (Roche, Germany) were applied to the eye sections to detect apoptosis. The sections were permeated with 0.1% Triton X-100 in 1% sodium citrate for 10 min, then they were incubated with a TUNEL reaction mixture for 1 h at 37 °C. The sections were observed under the confocal microscope, and all TUNEL-positive cells were counted.

### Cell culture and myosin 1f knockdown

We grew the immortalized murine microglial BV2 cell line because BV2 can be a good substitute for primary microglia in many experimental settings [25]. All cell lines were tested for mycoplasma contamination before use. Small interfering RNA (siRNA) of myosin 1f (768: CCACAUCUACUACCAGCUUTT AAGCUGGUAGUAGAUGUGGTT; 1413: GCAGGAGGAGUAUGUGCAATT UUGCACAUACUCCUCCUGCTT; 2662: GCGGACAGCUUCUUAGAAATT UUUCUAAGAAGCUGUCCGCTT) and TransIT-2X (MIR 6000, Mirus) were chosen for the knockdown of myosin 1f. For stimulation, 100 ng/ml of lipopolysaccharide (LPS, L2880, Sigma Aldrich, St. Louis, USA) was applied after 24 h.

### Western blot analysis

Retina and cell samples were lysed in a lysis buffer containing 50 mM Tris‐HCl (pH 8.0) and 0.1% SDS, as well as the complete Protease Inhibitor Cocktail (11697498001; Roche Applied Science), 150 mM NaCl, 1% Triton X‐100, and 1% sodium deoxycholate. We incubated the primary antibodies overnight. The antibodies were as follows: GAPDH (Proteintech, 60004‐1‐Ig, RRID: AB_2107436; Proteintech, Chicago, IL, USA), β-actin (Proteintech,20536-1-AP), AKT (C67E7, Rabbit mAb, CST), p-AKT (Ser473, D9E, XP, Rabbit mAb, CST), ERK1/2 (137F5, Rabbit mAb, CST), p-ERK1/2 (D13.14.4E, XP, Rabbit mAb), JNK (#9252, CST), p-JNK (81E11, Rabbit mAb #4668, CST), myosin 1f (1: 1000, Abcam, ab197215) (Santa Cruz, sc-376534).

### ELISA procedures

TNF-α and IL-1β were detected by using ELISA kits according to the manufacturer’s protocols (MTA00B, MLB00C, R&D Systems, Minneapolis, USA). The tissue samples were lysed in PBS, and the supernatant was collected. The cell samples were then collected from the cultural supernatant.

### Skeleton analysis and Sholl analysis

We used software Fiji (1.0) from https://imagej.net/Fiji/Downloads to complete the quantitative skeleton analysis [26]. The plugin of AnalyzeSkeleton was downloaded from https://imagej.net/Fiji/Downloadshttp://imagej.net/AnalyzeSkeleton. First, turn the image to 8-bit and convert it to grayscale. Adjust the brightness and contrast to make sure the microglia can be visualized. Click Despeckle toolbar to remove the noise of the image. Turn the image into binary by the Threshold toolbar. Skeletonize the image by clicking the toolbar of Skeletonize. Click the plugin AnalyzeSkeleton (2D/3D), then analyze it in Fiji by clicking the toolbar Skeleton/Analyze. We can get the average branch length and other data, such as the length of every branch, the endpoints voxel, the maximum branch length.

The Sholl analysis can plot the number of dendrite intersections against the radial distance from the soma center, which can also be performed by Fiji [27]. Adjust the brightness and contrast to make the image visualized. Download and install the plugin Simple Neurite Tracing and trace all the paths of microglia. Then click the Sholl analysis toolbar to calculate the intersection at a different distance from the soma.

### Quantitative real-time PCR

The primer sequences were acquired from Primerbank (bank: https://pga.mgh.harvard.edu/primerbank/), including TNF-α (F: CCCTCACACTCAGATCATCTTCT, R: GCTACGACGTGGGCTACAG), IL-1β (F: GAAATGCCACCTTTTGACAGTG, R: TGGATGCTCTCATCAGGACAG), IL-6 (F: TAGTCCTTCCTACCCCAATTTCC, R: TTGGTCCTTAGCCACTCCTTC), IRF8 (F: CGGGGCTGATCTGGGAAAAT, R: CACAGCGTAACCTCGTCTTC), CD68 (F: TGTCTGATCTTGCTAGGACCG, R: GAGAGTAACGGCCTTTTTGTGA) and myosin 1f (F: CTTTCACTGGCAGAGTCACAA, R: ATGAAGCGTTTGCGGAGGTT).

### Photography and optical coherence tomography (OCT) in vivo

The mice were anesthetized before the operation. Fundus photography and optical coherence tomography were performed on eyes with dilated pupils. The equipments were purchased from Phoenix Research Labs, Inc. Systems (Phoenix, USA).

### Flow cytometry

Digest the retina tissue into single cells, and stained with Annexin V/PI (Annexin V-FITC Apoptosis kit (Beyotime)) according to the manufacturer’s instructions. We assessed fluorescence intensity by flow cytometry using CytoFLEX (Beckman Coulter).

### Data and statistical analysis

The statistical analysis was conducted using Prism8 software. The data are presented as mean ± SEM, unpaired Student’s *t* test, *P < 0.05, **P < 0.01, ***P < 0.001, ****P < 0.0001; *P* > 0.05 was regarded as insignificant.

## Results

### Myosin 1f is upregulated after retinal detachment

**Fig. 1:**
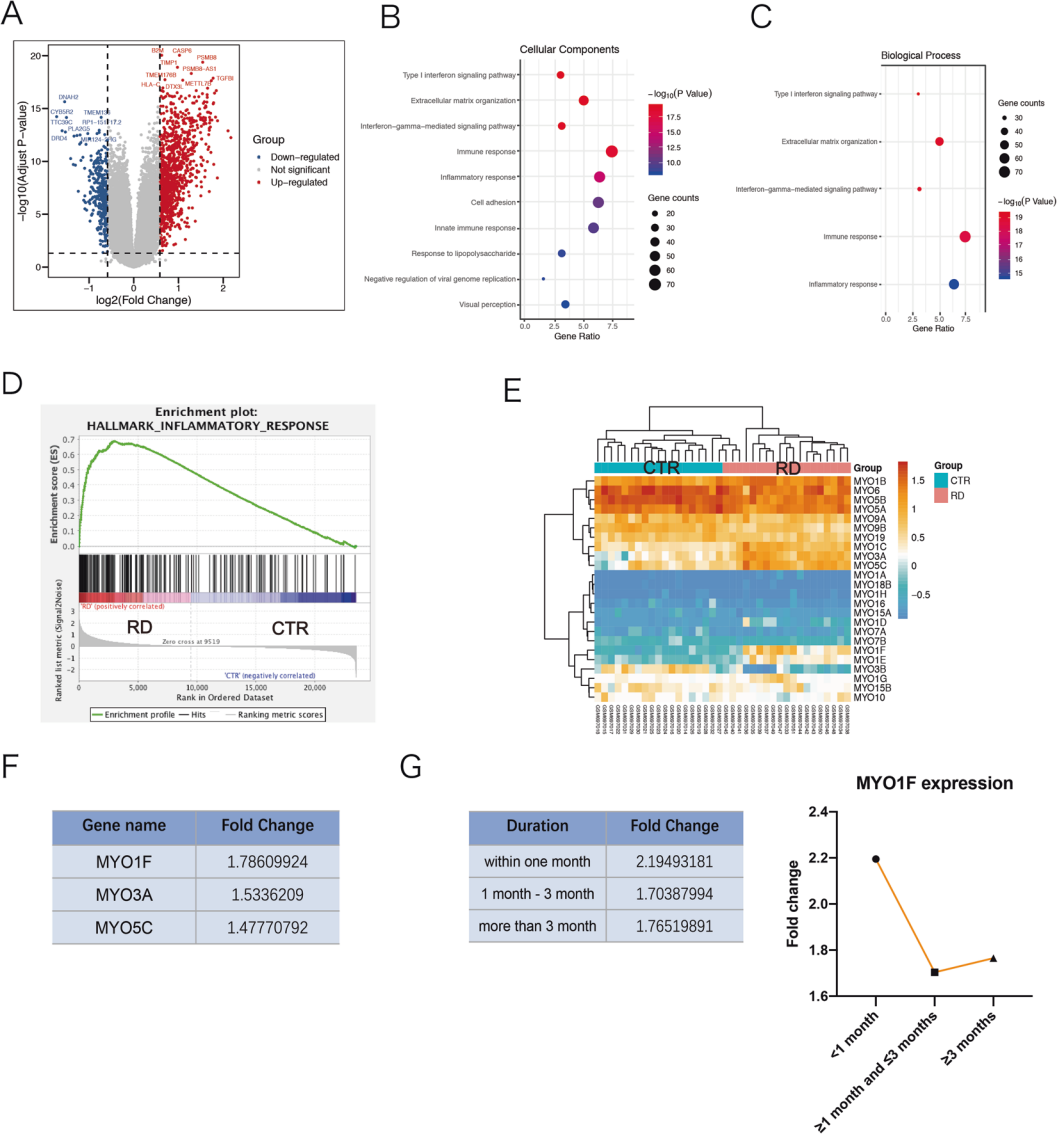
Transcriptome analysis of human retinal detachment. **A** Identification of differential expressed genes (DEGs) were set as |FC | > 1.5 and *P* < 0.05. Red dots on the volcano plot (**A**) represent upregulated genes while blue dots represent downregulated genes. **B**, **C** Gene ontology analysis. Cellular components analysis (**B**) and biological process analysis (**C**) showed possible functions of DEGs. **D** Gene-set enrichment analysis (GSEA) also revealed possible pathways possibly correlated to RD, including inflammatory response. **E**, **F** Expression pattern of myosins. Heatmap (**E**) demonstrated the most evaluated myosin, myosin 1f, with a FC = 1.786 (**F**). **G** The fold changes of at different RD duration.

We analyzed the data of GSE28133 from GEO datasets. Differential expression analysis revealed that 990 genes were upregulated and 272 genes were downregulated (|FC | > 1.5 and *P* < 0.05) (Fig. 1A). GO analysis indicated that immune response was involved in the pathology of RD (Fig. 1B, C). GSEA enrichment analysis also confirmed the results (Fig. 1D) (Supplementary 1, Fig. A). To discover the expression pattern of myosins after RD, a heatmap was examined and revealed that several myosins were upregulated, including MYO1F, MYO3A, and MYO5C, in the RD groups (Fig. 1E), and MYO1F was the most upregulated among the 3, with an FC of 1.786 (Fig. 1F). To figure out the expression pattern of MYO1F along with time, we divided the patients into three groups according to the RD duration, including within 1 month, 1 month to 3 months, and more than 3 months. The fold change MYO1F is the highest among the patients with a RD duration within one month (FC = 2.19493181) (Fig. 1G), suggesting an upregulation of MYO1F in the early phase.

**Fig. 2:**
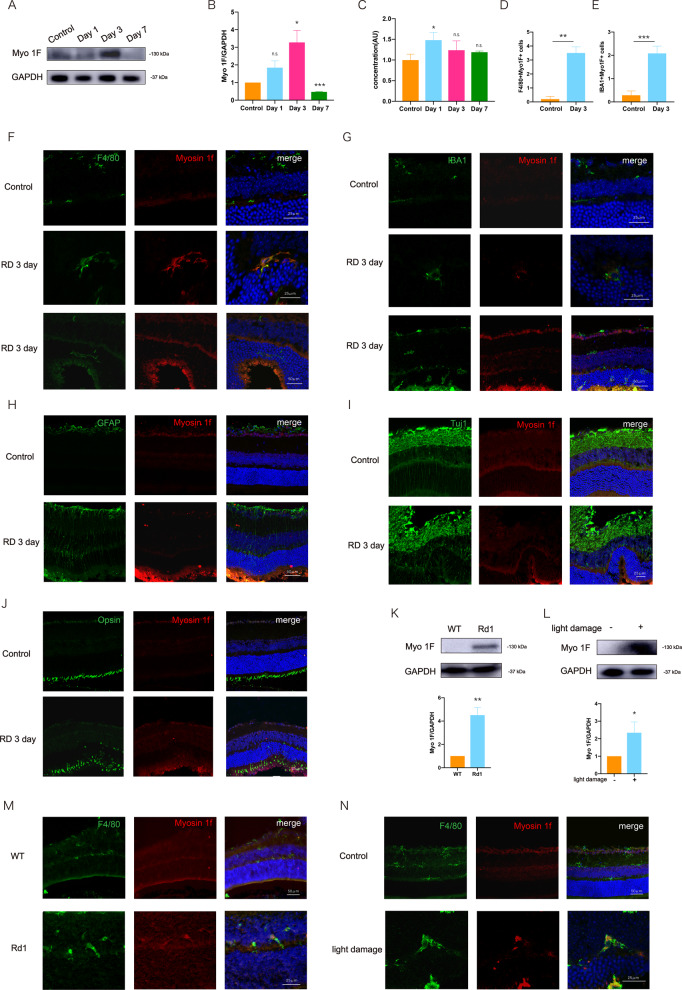
Myosin 1f is upregulated after the mouse model of retinal detachment. **A**, **B** The expression of myosin 1f at day 1, day 3, day 7 on western blot, it reached to peak at day 3 (**A**). The expression value is calculated by the optical density ratio of myosin 1f and GAPDH (**B**). **C** The expression of myosin 1f at day 1, day 3, day 7 on qPCR. **D** The count of F4/80 and myosin 1f-positive cells on eye sections under every scope (×40). **E** The count of IBA1 and myosin 1f-positive cells on eye sections under every scope (×40). **F** The Representative image of eye sections stained for F4/80 (green), myosin 1f (red), and dapi (blue). The eyeballs were taken down on day 3 after RD. **G** Representative image of eye sections stained for IBA1 (green), myosin 1f (red), and dapi (blue) (day 3). **H** Eye sections stained for GFAP (green), myosin 1f (red), and dapi (blue) (day 3). **I** Eye sections stained for Tuj1 (green), myosin 1f (red), and dapi (blue) (day 3). **J** Eye sections stained for Opsin (green), myosin 1f (red), and dapi (blue) (day 3). **K** Myosin 1f is also upregulated in *rd1* mouse day 7 after birth, quantification value is calculated by the optical density ratio of myosin 1f and GAPDH. **L** Myosin 1f is also upregulated in the light-injured retina (day 5), quantification value is calculated by the optical density ratio of myosin 1f and GAPDH. **M** Retinal sections of *rd1* mice (day 7 after birth) stained for F4/80 (green), myosin 1f (red), and dapi (blue). **N** Retinal sections of the light-injured retina (day 5) stained for F4/80 (green), myosin 1f (red), and dapi (blue). Data were presented as mean ± SEM, unpaired *t* test, *P < 0.05, **P < 0.01, ***P < 0.001, ****P < 0.0001.

To verify the results of the RNA sequencing, we detected the expression of myosin 1f in mouse retina samples. A western blot revealed that myosin 1f was upregulated on the detached retina, and it reached the peak at day 3 after detachment (Fig. 2A, B). qPCR results revealed that the transcription level of myosin 1f reaches to the peak at day 1 after the retinal-detachment model, earlier than protein expression (Fig. 2C). It is reasonable that the transcription is earlier than the protein translation and expression.

We examined the location of myosin 1f via immunofluorescence. Immunofluorescence confirmed that myosin 1f was upregulated within the retinal section after RD (Fig. 2D, E), and it was co-located with microglia markers, including F4/80 and IBA1 (Fig. 2F, G). Thus, myosin 1f is specifically expressed in IBA1 and F4/80-positive cells, since there was no sign of co-location between myosin 1f and GFAP (Fig. 2H), myosin 1f and Tuj1 (Fig. 2I), myosin 1f and Opsin (Fig. 2J).

*Rd1* mouse is a model for retinitis pigmentosa, which is also characterized by photoreceptor death and microglia activation [28]. Light damage is another model of photoreceptor degeneration [29, 30]. We discovered the same expression pattern of myosin 1f in both the *rd1* mouse model (Fig. 2K) and the light damage model (Fig. 2L). Similarly, myosin 1f was expressed on F4/80-positive cells within the retina sections of the *rd1* mouse model (Fig. 2M) and the light-damaged eye (Fig. 2N).

### Myosin 1f deficiency protects against photoreceptor death

To further study the function of myosin 1f, we investigated its effect on photoreceptor death in a myosin 1f−/− mouse model [18, 31, 32]. To verify that myosin 1f deficiency does not contribute to retinal injury and photoreceptor death, we observed the structure and function of the retina. Twenty-week-old myosin 1f−/− mice were compared with age-matched wild-types and showed normal structures on both optical coherence tomography (Supplementary 4, Fig. A, B) and HE section (Supplementary 4, Fig. C, D), and the amplitude of ERGs for both groups was within the normal range (Supplementary 4, Fig. E).

**Fig. 3:**
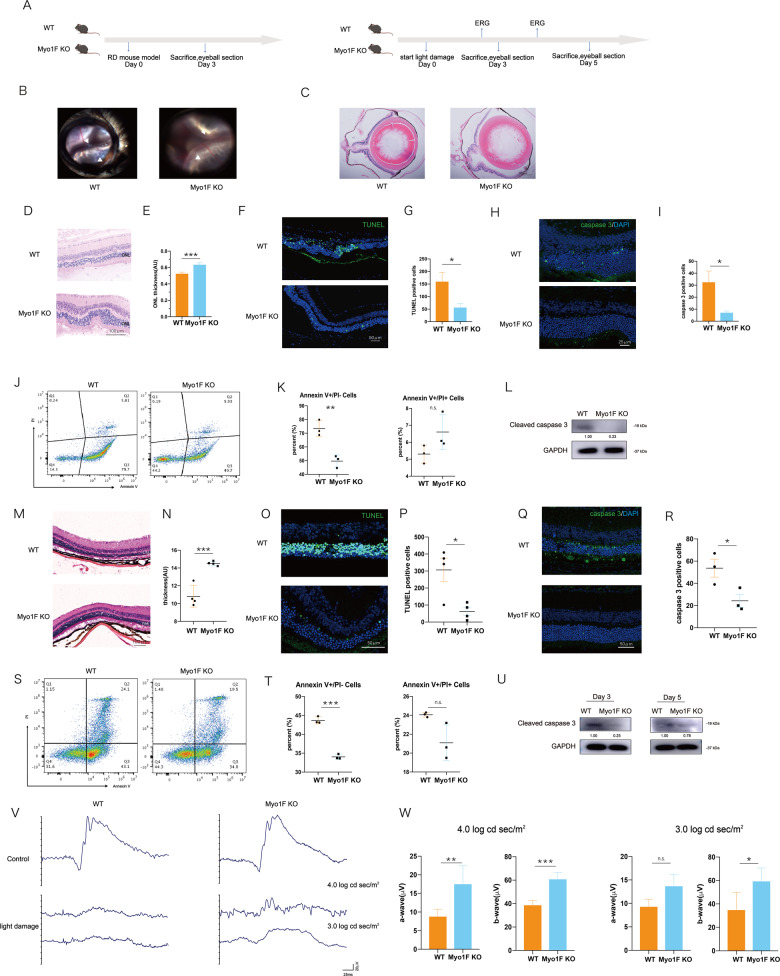
Observation of myo1f KO mice after retinal-detachment model and light damage after RD model. **A** Two groups of mice, myo1f KO and WT, were sacrificed on day 3 after RD. Two groups of mice were sacrificed on day 3 and day 5 after light damage. **B**, **C** Fundus photography (**B**) and HE (**C**) showed detachment of RPE and photoreceptor (day 3). **D**, **E** HE staining showed the thickness of ONL on the detached retina (day 3) of two groups (**D**). Scale bar, 100 μm. Measurements of ONL were taken by image J (**E**) (WT group *n* = 5, myo1f KO group *n* = 5). **F**, **G** Representative TUNEL staining in ONL (in green) (3 days after induction of RD) showed apoptosis of photoreceptors, scale bar, 50 μm (**F**), quantification of TUNEL-positive cells in ONL (**G**) revealed the significance (*n* = 5). **H**, **I** Representative cleaved caspase 3 staining in ONL (in green) (3 days after induction of RD) also showed apoptosis of photoreceptors, scale bar, 25 μm (**H**), quantification of cleaved caspase 3-positive cells in ONL (**I**) revealed the significance (*n* = 5). **J**, **K** Representative flow cytometry figure of Annexin V/PI staining (**J**), and quantification of the proportion of Annexin V + and Annexin V/PI + (**K**) revealed the significance. **L** Western blot of cleaved caspase 3. The expression value is calculated by the optical density ratio of cleaved caspase 3 and GAPDH. **M**, **N** HE staining showed the thickness of ONL on the light-injured retina (day 3) of two groups (**M**). Measurements of ONL were taken by image J (N (WT group *n* = 4, myo1f KO group *n* = 4). **O**, **P** Representative TUNEL staining in ONL (in green) (3 days after induction of light damage) showed apoptosis of photoreceptors, scale bar, 50 μm (**O**), quantification of TUNEL-positive cells in ONL (**P**) revealed the significance (*n* = 4). **Q**, **R** Representative cleaved caspase 3 staining in ONL (in green) (3 days after induction of light damage) also showed apoptosis of photoreceptors, scale bar, 50 μm (**Q**), quantification of cleaved caspase 3-positive cells in ONL (**R**) revealed the significance (*n* = 3). **S**, **T** Representative flow cytometry figure of Annexin V/PI staining after light damage (**S**), and quantification of the proportion of Annexin V + and Annexin V/PI + (**T**) revealed the significance. **U** Western blot of cleaved caspase 3 at day 3 after light damage. The expression value is calculated by the optical density ratio of cleaved caspase 3 and GAPDH. **V**, **W** ERG of two groups after light damage (**V**) and quantification of a wave and b wave at 4.0 log cd sec/m^2^ and 3.0 log cd sec/m^2^ (**W**). Data were presented as mean ± SEM, unpaired *t* test, *P < 0.05, **P < 0.01, ***P < 0.001, ****P < 0.0001.

We chose the RD model and light damage model as representative models of photoreceptor degeneration. Then we observed the myosin 1f−/− mice at 3 days after RD because it is reported that the apoptosis of photoreceptors is most extensive on the 3rd day. We also observed the phenotype of myosin 1f−/− mice in the light damage model on day 3 and day 5 (Fig. 3A). Fundus photography and HE staining showed that the retina remained detached on the 3rd day (Fig. 3B, C). To assess photoreceptor loss, we calculated the thickness of the outer nuclear layer (ONL) (Fig. 3D). Myosin 1f−/− mice exhibited thicker ONLs after RD (Fig. 3E). In addition, there were fewer TUNEL-positive cells in the myosin 1f−/− (Fig. 3F, G), which suggests that myosin 1f deficiency can protect against photoreceptor apoptosis. Cleaved caspase 3 is the activated caspase 3, which can be a marker of apoptosis [33, 34]. The count of cleaved caspase 3-positive cells is less in the myosin 1f−/− group compared to WT on the cleaved caspase 3 staining (Fig. 3H, I). Flow cytometry of Annexin V/PI further confirms the protective effect of myosin 1f deficiency (Fig. 3J, K), the Annexin V-positive cells were less in the myosin 1f−/− group. The expression of cleaved caspase 3 in western blot was lower in the myosin 1f−/− group (Fig. 3L).

In the light damage model, swe observed the ERG and apoptosis in the retina on day 3 and day 5. The ONL thickness was thinner in THE myosin 1f−/− group (Fig. 3M, N). In addition, there were fewer TUNEL-positive cells (Fig. 3O, P) and cleaved caspase 3-positive cells in the myosin 1f−/− group (Fig. 3Q, R) on day 3. Annexin V-positive cells were also less in the myosin 1f−/− group (Fig. 3S, T) at day 3. However, the difference of TUNEL-positive cells at day 5 (Supplementary 5, Fig. A, B) was not significant, the same as the percentage of Annexin V-positive cells (Supplementary 5, Fig. C, D). The disappearance of difference at day 5 may be due to the severe damage in the retina of both groups. Western blot results of cleaved caspase 3 showed a distinct difference between the two groups at day 3, while the less difference at day 5 (Fig. 3U). The amplitude of a wave and b wave on ERG were stronger at 3.0 and 4.0 log cd sec/m^2^ in myosin 1f−/− group at day 3 (Fig. 3V, W). The ERG signal was almost extinguished at day 5 in both groups, which suggests the severe damage in the retina (Supplementary 5, Fig. E).

### Myosin 1f influences the activation of IBA1-positive cells

**Fig. 4:**
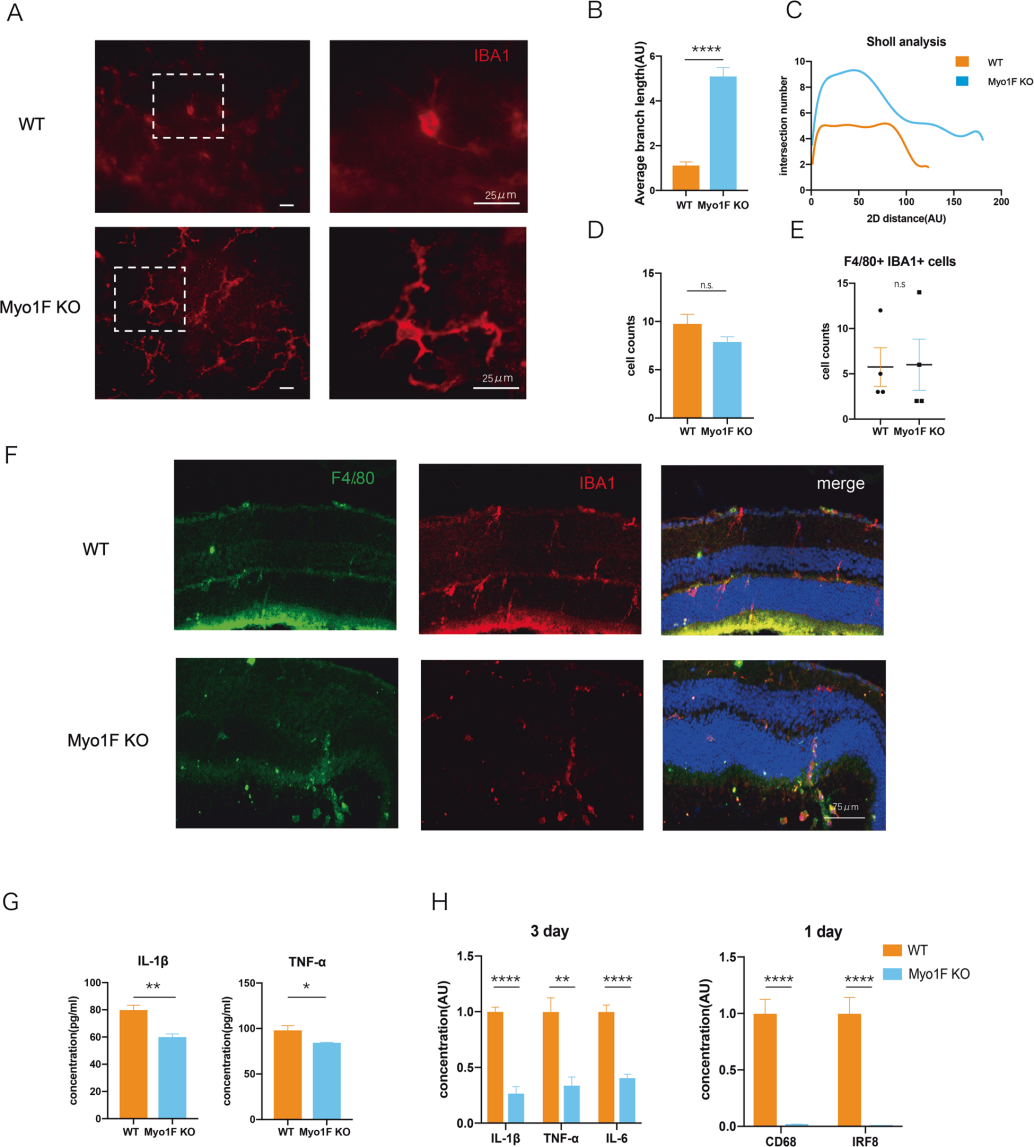
Knockout of myosin 1f affects the function of microglia. **A** Flatmount of the detached retina of WT and myo1f KO mice, stained by IBA1 (in red), showed the morphology of microglia. Scale bar, 25 μm. **B**, **C** Skeleton analysis (**B**) and Sholl analysis (**C**) were conducted to quantify microglia morphology. The less average branch length is, the more activated microglia is. Similarly, the less interaction number is, the more activated microglia is. **D** IBA1 + cell counts of the stretched preparation of retina after retinal detachment (*n* = 8). **E** The cell count of both F4/80 + IBA1 + cells infiltrated into the outer nuclear layer (*n* = 4). **F** Representative F4/80 + (green) IBA1 + (red) staining in ONL (3 days after induction of RD). **G** ELISA analysis of IL-1β and TNF-α in the detached retina of WT and myo1f KO mice (day 3). The quantification of total protein was 0.4 μg/ml. **H** QPCR analysis of IL-1β and TNF-α in the detached retina at day 3, CD68 and IRF8 at day 1. Data were presented as mean ± SEM, unpaired *t* test, *P < 0.05, **P < 0.01, ***P < 0.001, ****P < 0.0001.

We analyzed the morphology of IBA1-positive cells 3 days after RD via stretched preparation and immunofluorescence. The cells were more extended and had more branches in the retina of myosin 1f−/− mice, whereas the microglia of the WT retina were more shrunken and tended to be round (Fig. 4A). A quantitative skeleton analysis and a Sholl analysis showed more intersections in the myosin 1f−/− group (Fig. 4B, C). This indicates that myosin 1f may contribute to microglia activation. We have also counted the IBA1 + cells of the stretched preparation and cell counts on the retina of myosin 1f−/− showed no difference (Fig. 4D). Microglia can migrate to injury sites after RD, especially the outer nuclear layer (ONL), however, we have not observed the difference in numbers of infiltrated microglia (Fig. 4E, F).

IL-1β, IL-6, and TNF-α are classic pro-inflammatory cytokines in activated microglia [35]. The ELISA analysis suggests IL-1β and TNF-α (Fig. 4G) were also downregulated in myosin 1f−/− mice at day 3. The mRNA expression is in accordance with the protein expression pattern. We observed the downregulation of IL-1β, TNF-α, and IL-6 in myosin 1f–/− mice at day 3, compared to WT mice. In addition, CD68 and IRF8, markers related to the degree of activation [36, 37], were lower at day 1 in myosin 1f−/− mice (Fig. 4H).

### Elimination of microglia can abolish the protective effect of myosin 1f deficiency

**Fig. 5:**
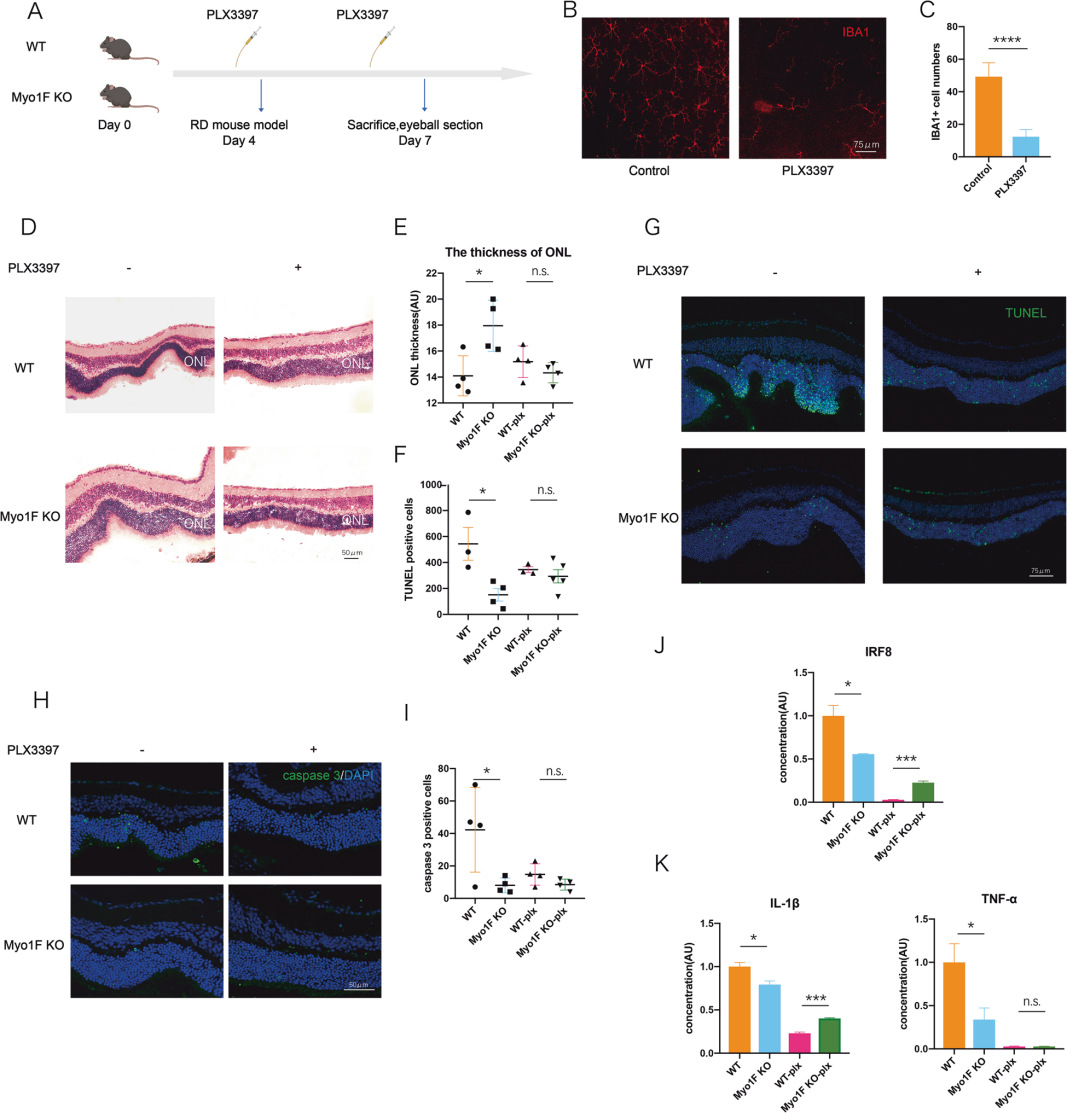
Elimination of microglia reverses the protective effect of myosin 1f deficiency. **A** PLX3397 was given to WT and myo1f KO mice every day from day 1 to day 6, the mice were sacrificed on day 7, 3 days after the mouse model of retinal detachment (day 4). **B**, **C** Representative image of flatmount of the detached retina (**B**) and elimination efficacy (**C**). **D**, **E** Representative HE staining showed the thickness of ONL on the detached retina (**D**) (scale bar, 50 μm) and quantification of ONL thickness (**E**) (*n* = 4). **F**, **G** Representative TUNEL staining (**F**) (in green) (3 days after induction of RD) (scale bar, 75 μm) and quantification of TUNEL-positive cells in ONL (**F**) among four groups showed apoptosis of photoreceptors after microglia elimination. **H**, **I** Representative cleaved caspase 3 staining (**H**) (in green) (3 days after induction of RD) (scale bar, 50 μm) and quantification of cleaved caspase 3-positive cells in ONL (**I**) among four groups showed apoptosis of photoreceptors after microglia elimination. **J**, **K** qPCR analysis demonstrated the fold change of IRF8, IL-1β, and TNF-α (day 3). Data were presented as mean ± SEM, unpaired *t* test, *P < 0.05, **P < 0.01, ***P < 0.001, ****P < 0.0001.

To verify that myosin 1f deficiency can protect photoreceptors by regulating the activation of microglia, we administered PLX3397 [38] to mice via oral gavage to eliminate mononuclear phagocytes, including microglia. PLX3397 is an inhibitor of the CSF1R receptor, which is widely expressed in mononuclear phagocytes [39]. It is reported that PLX3397 can reduce tissue macrophages without affecting myeloid cells [40]. We started gavage on day 1, and a dose was delivered every day until day 7. We conducted the RD model experiment on day 4 and observed on day 7 (Fig. 5A). Another two groups without PLX3397 gavage were set as matched groups. PLX3397 gavage significantly reduced microglia number according to the IBA1-positive cells on the retina (Fig. 5B, C).

The retinal thickness and the TUNEL-positive cells were calculated to evaluate photoreceptor death. The ONL was thicker in the WT group compared to the myosin 1f−/− group without PLX3397, the same as the results in Fig. 3; While there was no difference in ONL thickness between the WT and myosin 1f−/− group with PLX3397 (Fig. 5D, E). Likewise, there were more TUNEL-positive cells in the WT group compared to the myosin 1f−/− group without PLX3397, while there were no significant differences in TUNEL-positive cell counts between the two groups with PLX3397 (Fig. 5F, G). The cleaved caspase 3 staining demonstrated the same results. There were more cleaved caspase 3-positive cells in the WT group compared to the myosin 1f−/− group without PLX3397, while there were no significant differences in cleaved caspase 3-positive cell counts between the two groups with PLX3397 (Fig. 5H, I).

We then detected mRNA expression after microglia elimination. Interestingly, IRF8 expression even reversed after PLX3397 (Fig. 5J). Similarly, the mRNA expression of IL-1β and TNF-α also disappeared or reversed (Fig. 5K). That is to say, microglia elimination abolished the protective effect of myosin 1f deficiency.

### Myosin 1f affects microglia activation by regulating the MAPK/ AKT pathways

**Fig. 6:**
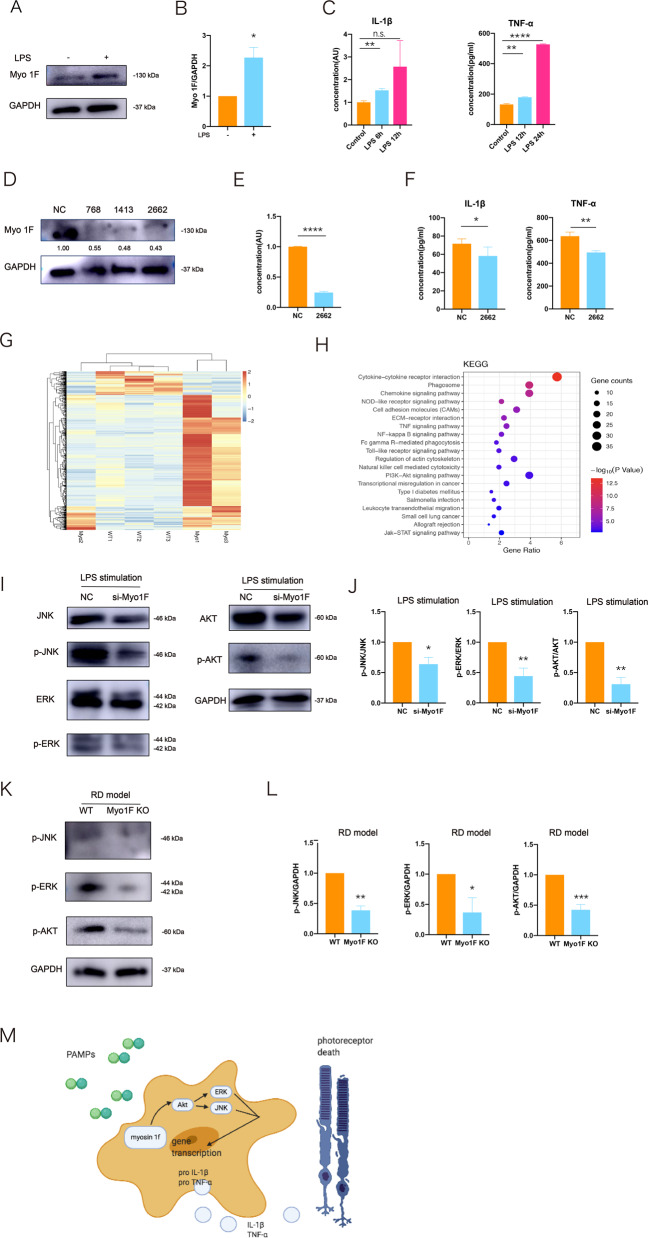
Myosin 1f affects the activation of microglia by regulating AKT and MAPK pathways. **A**, **B** In vitro, western blot showed that myosin 1f is upregulated after stimulated by LPS (100 ng/ml) for 24 h (**A**), the expression value is calculated by the optical density ratio of myosin 1f and GAPDH (**B**). **C** The expression of IL-1β (qPCR) and TNF-α (ELISA) at every time point. **D**, **E** After siRNA on BV2 cell lines, we detected the expression of myosin 1f by western blot (**D**). Three sequences of siRNA (768,1413,2772) were designed to knock down myosin 1f, 2662 were chosen to further confirm the efficiency (**E**). **F** ELISA analysis of IL-1β and TNF-α after knockdown myosin 1f. **G** Heatmap of RNA-seq from the detached retina of WT and myosin 1f−/− mice. **H** KEGG analysis of RNA-seq. **I**, **J** In vitro, western blot analysis showed an expression pattern of AKT and MAPK pathways (**I**). All the quantification of phosphorylation protein is calculated by the optical density ratio of phosphorylation protein and its corresponding protein (**J**). **K**, **L** In vivo, western blot analysis showed an expression pattern of AKT and MAPK pathways (**K**). All the quantification of phosphorylation protein is calculated by the optical density ratio of phosphorylation protein and GAPDH (**L**). **M** Graphical abstract of the effect of myosin 1f in the activation of microglia. Data were presented as mean ± SEM, unpaired *t* test, *P < 0.05, **P < 0.01, ***P < 0.001, ****P < 0.0001.

To further explore the molecular mechanism underlying myosin 1f-mediated photoreceptor degeneration, we used lipopolysaccharide (LPS) to stimulate BV2 cell lines in vitro. BV2 is an immortalized cell line derived from mice [41]. We noticed the upregulation of myosin 1f after stimulation (Fig. 6A, B). The transcription level of IL-1β rose 6 h after stimulation. In addition, an ELISA analysis suggests that TNF-α rose after LPS stimulation (Fig. 6C). Furthermore, we developed a siRNA knockdown system for myosin 1f. We designed three sequences of siRNA, including 768, 1413, and 2662; all of them led to a significant decrease of myosin 1f at the protein level (Fig. 6D). We chose 2662 to detect the quantitative efficiency of the siRNA, and the efficiency reached about 80% (Fig. 6E).

We used LPS to stimulate the control and myosin 1f knockdown cells. Then we detected the expression of IL-1β and TNF-α in the cellular supernatant at hour 24 via ELISA, and we found that both cytokines went down in the knockdown group (Fig. 6F).

Transcriptional analysis has identified 673 DEGs involved in the biology process of myosin 1f-mediated photoreceptor degeneration (Fig. 6G). KEGG analysis indicates TNF signaling pathway, NF-kappa B signaling pathway, PI3K-AKT signaling pathway are possibly related to the microglia activation (Fig. 6H). Previous studies also suggested that MAPK and AKT signaling pathways were implicated in the activation of microglia [42, 43]. TNF signaling pathway is closely related to MAPK, AKT, and NF-kappa B signaling pathways through the activation of two receptors [44–47]. We then detected the expression of proteins related to MAPK and AKT. The proportion of phospho-AKT decreases after 24 h of LPS stimulation (Fig. 6I, J). As the two main components of MAPK, phospho-ERK, and phospho-JNK also showed significant decreases (Fig. 6I, J). In addition, we repeated western blot to detect the phosphorylation protein in the detached retina of WT and myosin 1f−/− mice (Fig. 6K, L). In vivo results may further verify the consequences of myosin 1f regulation of microglia activity through the MAPK and AKT pathways.

## Discussion

In this study, we have discovered that myosin 1f was significantly upregulated after photoreceptor degeneration in both the human retina and mouse model. We further demonstrated that myosin 1f can regulate microglia activation, whereas the absence of myosin 1f can protect photoreceptors by inhibiting the MAPK and AKT pathways and decreasing the expression of inflammatory cytokines, such as TNF-α and IL-1β, in microglia (Fig. 6M).

The vision loss after RD is mainly due to photoreceptor death [48]. Various forms of cell death are involved in this pathology, including apoptosis, necrosis, and autophagy, which peak at 2–3 days after RD [49]. No current techniques can stop photoreceptor death completely [6]. This indicates that cell death is a complicated process involving different pathologies, including inflammation. The sequence of the human retina sample after RD demonstrated that immune response and neurodegeneration are two major biological processes involved in RD [7].

Besides, we have discovered that myosin 1f was upregulated after photoreceptor degeneration. Myosin 1f, a class 1 myosin, can regulate the immune response [32, 50, 51]. We found that myosin 1f was the most upregulated myosin by re-analyzing human RD sequence data, and we verified the results in a mouse RD model. MYO1F is upregulated at the early phase and downregulated at day 7, the protein expression is even lower than the baseline according to Fig. 2B. It remains unclear that the specific mechanism of myosin 1f regulation, how it rises, and how it reduces. Our data suggested myosin 1f is involved in the early damage of photoreceptors, indicating the importance of intervention time when choosing myosin 1f as a therapeutic target in photoreceptor degeneration.

We also found that myosin 1f is generally upregulated in *rd1* mutation mouse model and light-induced retinal injury. The *rd1* mouse is a classical model for retinitis pigmentosa [52]. Microglia are activated and express inflammatory cytokines in the *rd1* mouse model retina. A similar pathology occurs in light-induced injury. Thus, we discovered that myosin 1f was also upregulated in the two latter cases, which suggests it could play a key role in photoreceptor degeneration. It is reported that myosin 1f is also upregulated in brain neurodegeneration, such as Alzheimer’s disease (AD), Huntington’s disease (HD), and Parkinson’s disease (PD) [53], suggesting the upregulation of myosin 1f is a common phenomenon in neuroinflammation.

We also discovered that myosin 1f is highly expressed in microglia. Via immunofluorescence, we found that myosin 1f displayed a strong co-localization with mononuclear macrophages, indicating a possible relationship between myosin 1f and microglia activation after retinal injury. Interestingly, myosin 1f can also regulate the M1 polarization by stimulating intercellular adhesion in macrophages via AKT, STAT3, and NF-κb pathways [18]. Microglia originates from the yolk sac, it evolves from yolk sac macrophages, and colonizes the embryonic central nervous system [54, 55]. It is not surprising that microglia and macrophage have many characteristics and functions in common. Our study provides further evidence that myosin 1f plays an important role in mononuclear phagocytes activation.

To further determine the function of myosin 1f, we developed a myosin 1f KO mouse model, and it showed a significant reduction in neuron death. It suggested that myosin 1f deficiency could be protective to neurons and it is possible to work by affecting the activation of microglia.

Microglia, as a resident immune cell, is among the main effector cells of neuroinflammation after retinal injury [56]. In our study, we have discovered that myosin 1f deficiency has reduced the expression of inflammatory cytokines such as TNF-α and IL-1β. Besides, we found the morphological difference of microglia in myosin 1f KO retina and WT retina after RD. After the elimination of microglia, the protective effect of myosin 1f KO disappeared. Therefore, we think myosin 1f can regulate the activation of microglia and involved in neuroinflammation, which leads to the death of photoreceptors. By eliminating microglia, we want to emphasize that microglia have to exist first, then it could be regulated by myosin 1f. In fact, microglia is a double-edged sword [57]. Although overactivated microglia can release cytotoxic factors that lead to neuron death [54, 58], it can also protect neurons by secreting neuroprotective factors and phagocytosing injured cells [59]. The state of microglia can be decided by the course of the disease, the immune microenvironment, and interactions with other cells. Still and all, the two states of microglia are dynamic and coexist. Discovering a way to inhibit the inflammation pathway is still a promising target for retinal degeneration.

We discovered that myosin 1f can regulate the MAPK and AKT pathways to promote the transcription of pro-inflammatory cytokines and activate microglia. It is known that MAPK and NF-κb pathways are crucial in the release of microglia pro-inflammatory cytokines. MAPK can enhance the activation of NF-κb in LPS-induced BV2 microglia. The inhibitor of MAPK/NF-κB signaling pathways, such as sulforaphane [42], SB203580, and PDTC can downregulate microglia-mediated neuronal damage [60]. Mitogen-activated protein kinases (MAPK) signal the transduction pathway, which consists of ERKs, c-Jun NH2-terminal kinases (JNKs), and p38 MAPKs and is reported to be related to inflammation, cell proliferation, and apoptosis [61, 62]. It is also associated with microglia-induced neuroinflammation and the secretion of neurotoxic cytokines [63, 64]. AKT is an important protein of signaling transduction, which is involved in multiple pathways [16], and it can promote the expression of pro-inflammatory cytokines [43]. AKT can also be an upstream regulator of the NF-κb pathway [65]. NF-κb is a classic, crucial transcription factor in both innate and adaptive immune responses, and it participates in microglia-induced neuroinflammation [42, 66]. Our results reveal that myosin 1f could be the common upstream of MAPK and AKT pathways, making it a promising target for neuroprotective approaches.

The Myosin family, as a component of the cytoskeleton, was reported to be involved in several kinds of biological processes, including muscle contraction, intracellular transport, tethering, signaling, cell division, and cytoskeleton organization [67]. Myosins can influence signaling through phosphorylation, receptor recycling, interaction with integrin, and so on [68, 69]. Class II myosin can increase the integrin β1 activity by clustering, and integrin β1 is required for the activation of the AKT pathway [69]. Myosin 1C, myosin 1E, and myosin 1G can regulate TGF-β signaling by regulating the recycling and redistribution of TGF-β receptors to the cell membrane. Myosin 1f may work in a similar way. However, more future researches are needed to explore the interaction of myosin 1f and downstream pathways.

Myosin 1f can help neutrophil to transform and migrate to injury sites, as it consists of a motor domain with an actin-binding site, which enables the force transmission to nuclear and enables nuclear to transform [31]. Besides, it is reported that myosin 1f enhances intracellular adhesion through regulating the mobilization and stability of αVβ3 integrin [18]. Though myosin 1f is involved in the motility of neutrophils [31], it is surprising that myosin 1f does not affect the migration of microglia to the injury site. Instead, it affects the morphology of microglia, suggesting that the function of myosin 1f could be various among different immune cell types. The chemotaxis and migration of microglia are controlled by numerous signaling pathways and key molecules, such as PI3K, PLA2, PKA, Src family kinase, and myosin families [70]. It is reported that non-muscle myosin II enhances migration and phagocytosis by regulating the activity of myosin light chain kinase (MLCK) and interaction with actin [71–73]. In summary, class II myosins are the probably main effector molecules in microglia migration instead of myosin 1f.

In this study, we have revealed the function of myosin 1f in neuroinflammation, through the regulation of microglia activation. We wondered that the upregulation of myosin 1f may be universal in other retinal degeneration models, such as light damage and the *rd1* mouse model. Our finding may provide a new perspective for neuroprotection in photoreceptor degeneration. It is reported that several compounds have been discovered as the inhibitors of myosins, such as pentachloropseudilin (PCLP), a pseudilin derivative, which is a class one myosin-specific inhibitor [51, 74]; Blebbistatin [75], a myosin-2 inhibitor; Azidoblebbistatin, a photoreactive myosin inhibitor [51]. Our data suggest that myosin 1f may be a novel pharmacological target for protecting photoreceptors and preserving visual acuity.

## Supplementary

supplementary figure legends
Supplementary figure 1
Supplementary figure 2
Supplementary figure 3
Supplementary figure 4
Supplementary figure 5
DEG dataset1
