# Supplementary material for: Myosin 1f-mediated activation of microglia contributes to the photoreceptor degeneration in a mouse model of retinal detachment
Source: Cell Death Dis. 2021 Oct 9;12(10):926. doi: 10.1038/s41419-021-03983-3 (PMC8502177; doi:10.1038/s41419-021-03983-3)
Supplement: Supplementary file 1 — supplementary figure legends [file 41419_2021_3983_MOESM1_ESM.docx]

**Supplementary Figure legends**

**Supplementary figure 1**

A. Gene-set enrichment analysis (GSEA) also revealed possible pathways possibly correlated to RD, including interferon-gamma response, IL-6-STAT3 signaling, complement, IL-2-STAT5 signaling.

**Supplementary figure 2**

Negative control of figure 2. A. Negative control of F4/80 and myosin 1f of both control and RD group. B. Negative control of IBA1 and myosin 1f of both control and RD group. C. Negative control of GFAP and myosin 1f of both control and RD group. D. Negative control of Tuj1 and myosin 1f of both control and RD group. E. Negative control of Opsin and myosin 1f of both control and RD group. F. Negative control of caspase 3 of control (without RD), WT and myo1f KO (after RD) group.

**Supplementary figure 3**

**A.** The WB of myosin 1f pull down after overexpression. The vector is pcDNA3.1 with Flag tag. **B.** Knockdown of myosin 1f. **C.** Repeated result of Figure 2A with another anti-MYO1F (santa cruz, sc-376534**) D.** Repeated result of Figure 2L with another anti-MYO1F (santa cruz, sc-376534**) E.** The expression expression of myosin 1f at day 7 of *rd1* mouse on qPCR. **F.** Repeated result of Figure 2O with another anti-MYO1F (santa cruz, sc-376534**) G.** The expression expression of myosin 1f at day 7 of mouse on qPCR. **H.** Repeated result of Figure 6A with another anti-MYO1F (santa cruz, sc-376534**) I.** Repeated result of Figure 6D with another anti-MYO1F (santa cruz, sc-376534**)**

**Supplementary figure 4**

Myosin 1f deficiency does not affect the structure and function of retina. A-D. OCT (A, B) and HE (C, D) staining reflects the thickness of WT and myo1f KO mouse retina. The scale bar in OCT is 120 $\mu m$. Data were presented as mean±SEM, unpaired t test, n.c. *P >*0.05. E. Electroretinogram of WT and myosin 1f mice, more than 20 weeks- year-old.

Data were presented as mean±SEM, unpaired t test, **P <* 0.05, ***P <* 0.01, ****P <* 0.001, *****P <* 0.0001.

**Supplementary figure 5**

**A-B.** Representative TUNEL staining (in green) of light damage (day 5) (scale bar, 100$\mu m$) (A) and quantification of TUNEL-positive cells in ONL (n=3) (B). **C-D.** Representative flow cytometry figure of Annexin V/PI staining (C) (day 5), and quantification of proportion of Annexin V+ and Annexin V/PI+ (D). **E.** ERG of two groups after light damage (day 5).
