# Supplementary figures and images for: Myosin 1f-mediated activation of microglia contributes to the photoreceptor degeneration in a mouse model of retinal detachment
Source: Cell Death Dis. 2021 Oct 9;12(10):926. doi: 10.1038/s41419-021-03983-3 (PMC8502177; doi:10.1038/s41419-021-03983-3)

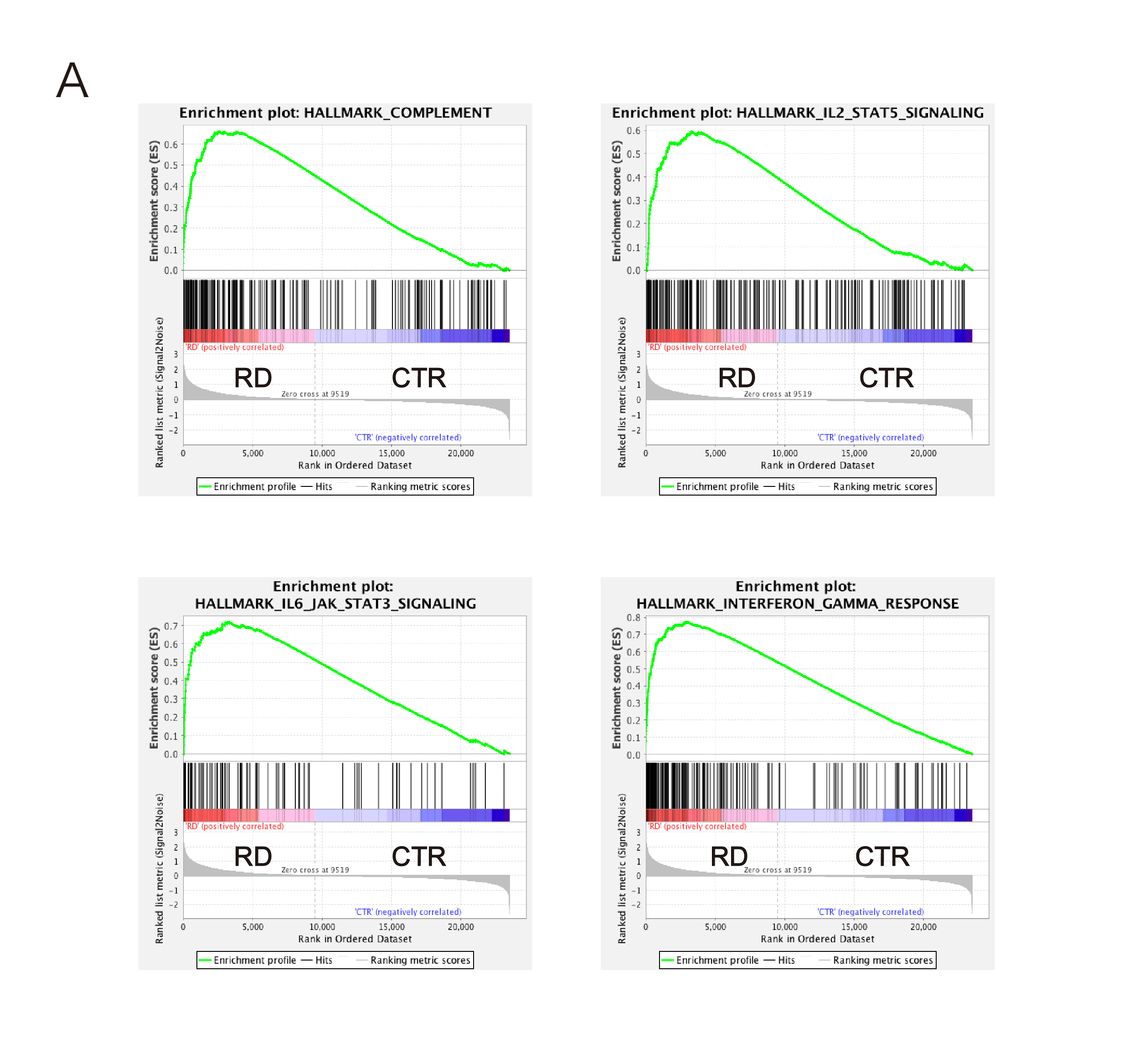

Supplement: Supplementary file 2 — Supplementary figure 1 [file 41419_2021_3983_MOESM2_ESM.png]

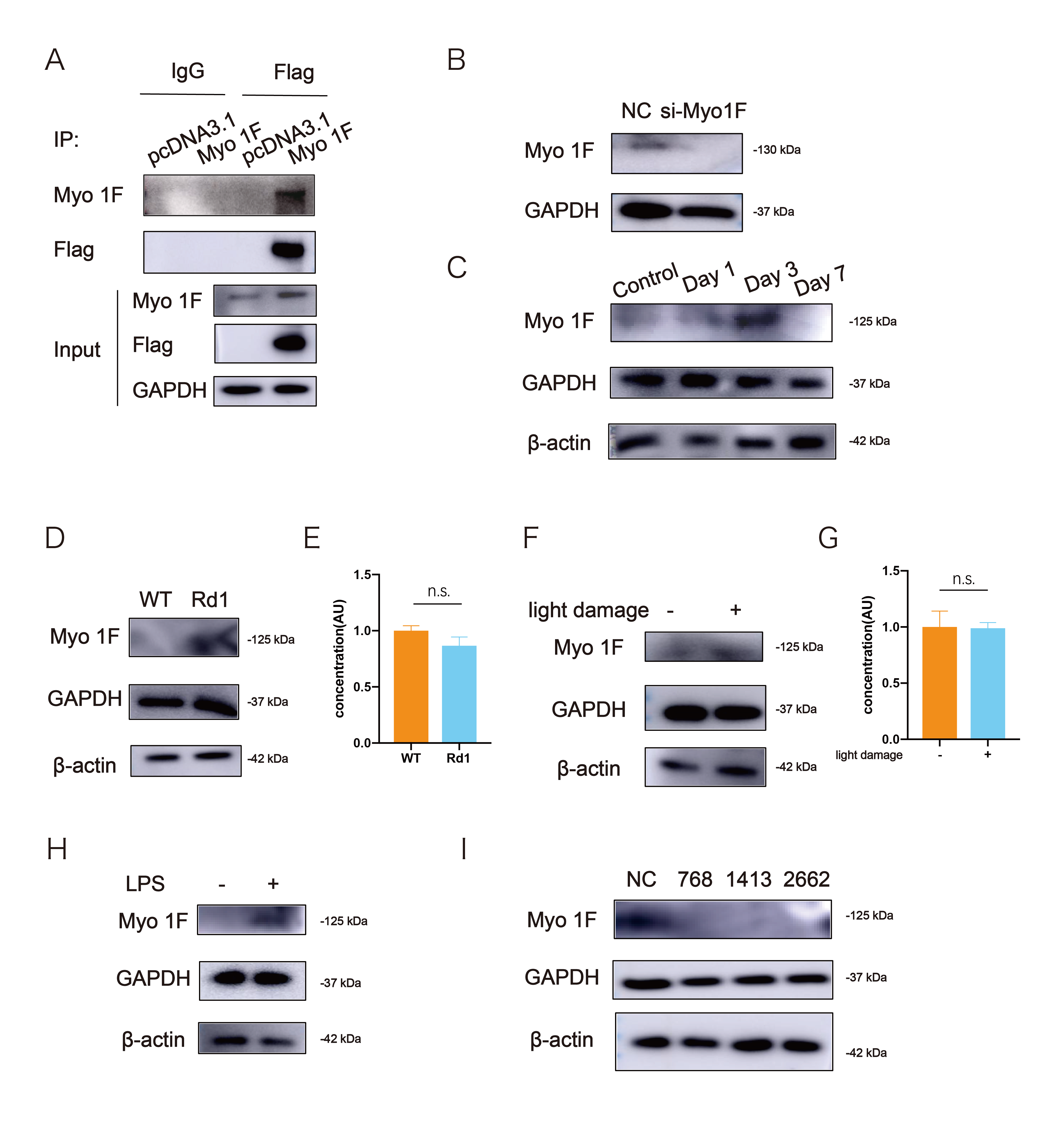

Supplement: Supplementary file 4 — Supplementary figure 3 [file 41419_2021_3983_MOESM4_ESM.png]

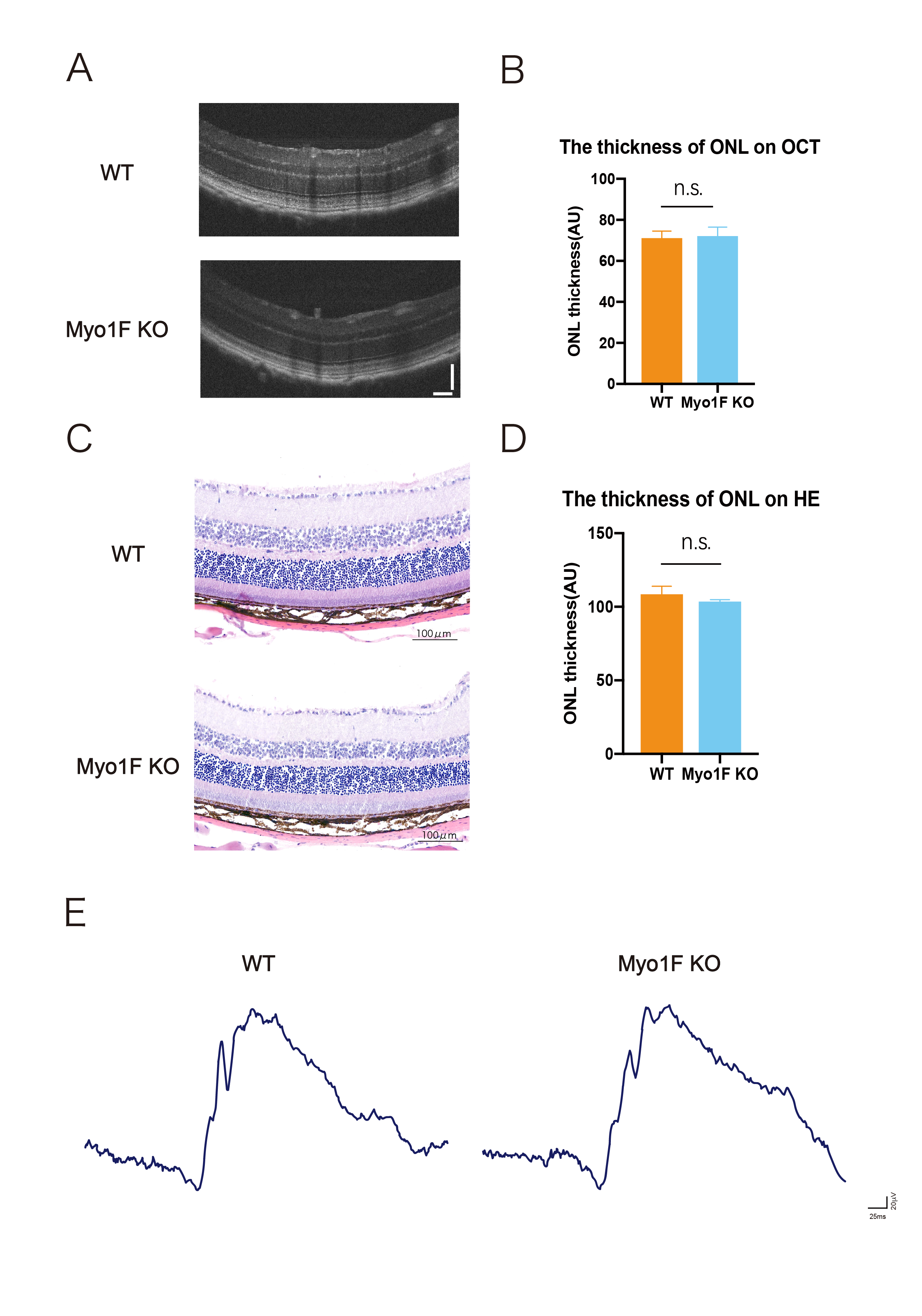

Supplement: Supplementary file 5 — Supplementary figure 4 [file 41419_2021_3983_MOESM5_ESM.png]

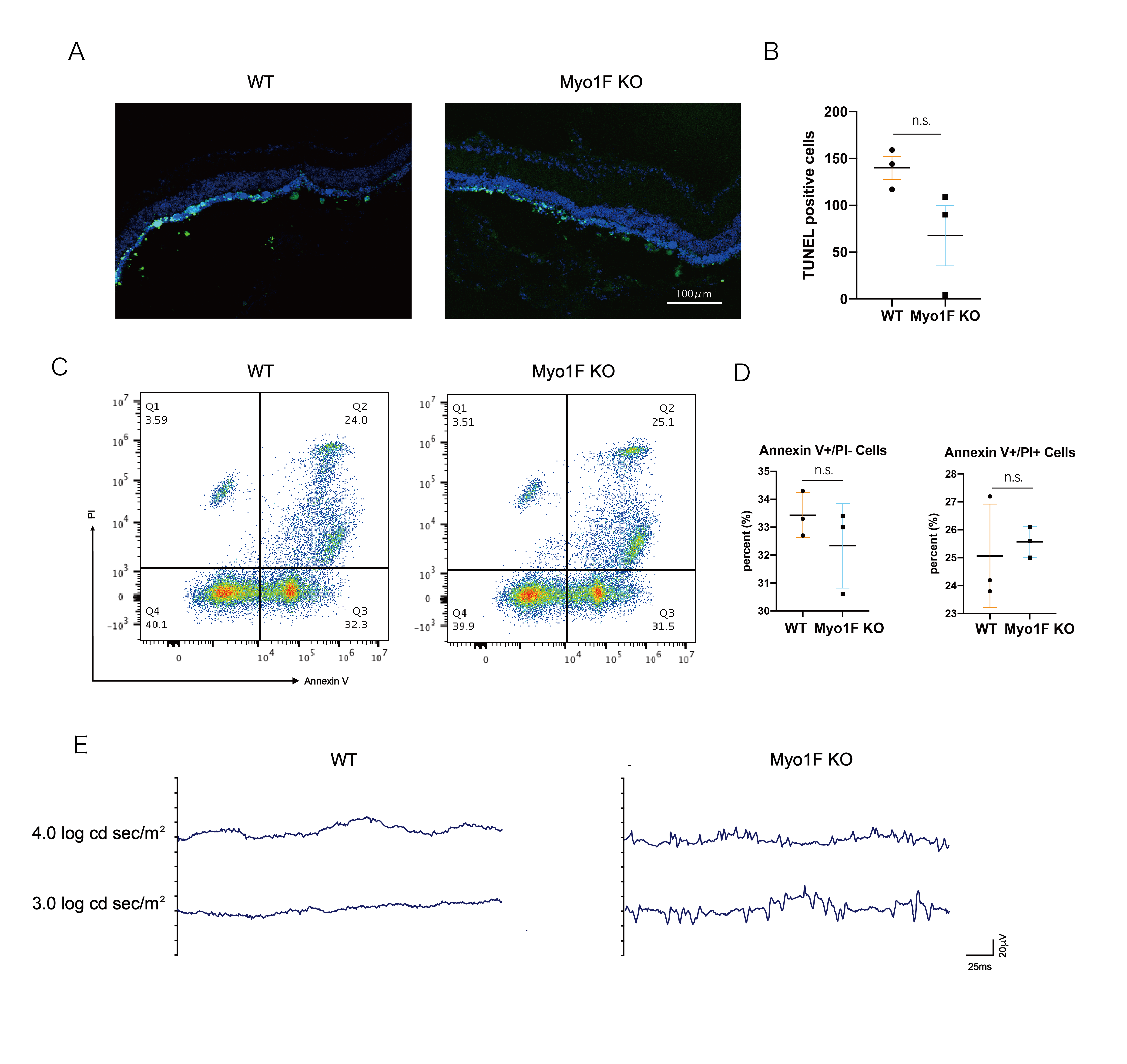

Supplement: Supplementary file 6 — Supplementary figure 5 [file 41419_2021_3983_MOESM6_ESM.png]
